# Bayesian methods outperform parsimony but at the expense of precision in the estimation of phylogeny from discrete morphological data

**DOI:** 10.1098/rsbl.2016.0081

**Published:** 2016-04

**Authors:** Joseph E. O'Reilly, Mark N. Puttick, Luke Parry, Alastair R. Tanner, James E. Tarver, James Fleming, Davide Pisani, Philip C. J. Donoghue

**Affiliations:** 1 School of Earth Sciences, University of Bristol, Life Sciences Building, Tyndall Avenue, Bristol BS8 1TQ, UK; 2 School of Biological Sciences, University of Bristol, Life Sciences Building, Tyndall Avenue, Bristol BS8 1TQ, UK

**Keywords:** parsimony, Bayesian, likelihood, phylogenetics, morphology

## Abstract

Different analytical methods can yield competing interpretations of evolutionary history and, currently, there is no definitive method for phylogenetic reconstruction using morphological data. Parsimony has been the primary method for analysing morphological data, but there has been a resurgence of interest in the likelihood-based Mk-model. Here, we test the performance of the Bayesian implementation of the Mk-model relative to both equal and implied-weight implementations of parsimony. Using simulated morphological data, we demonstrate that the Mk-model outperforms equal-weights parsimony in terms of topological accuracy, and implied-weights performs the most poorly. However, the Mk-model produces phylogenies that have less resolution than parsimony methods. This difference in the accuracy and precision of parsimony and Bayesian approaches to topology estimation needs to be considered when selecting a method for phylogeny reconstruction.

## 1. Introduction

Morphology once provided the only means of inferring evolutionary trees, but it was effectively rendered obsolete by molecular sequence data and the development of sophisticated molecular evolutionary models for phylogenetic analysis [1]. However, with the recognition that fossil species are integral to correctly inferring patterns of character evolution and changes in diversity, as well as in establishing evolutionary timescales, morphological data are enjoying a phylogenetic renaissance [2], allowing fossil species to be assigned to their correct branches in the Tree of Life. Methods for phylogenetic analysis of morphological data remain underdeveloped and though likelihood models are available that may more accurately accommodate the vagaries of morphological datasets [3], including high rates of heterogeneity and a preponderance of missing data [4], parsimony remains the method of choice, principally perhaps as a consequence of tradition. Indeed, a recent simulation-based study by Wright & Hillis [5] demonstrated that a Bayesian implementation of Lewis's Mk-model [3] strongly outperforms parsimony, especially when rates of character change are high, or when relatively few characters are analysed. The conclusions drawn by Wright & Hillis [5] were based on data effectively simulated using the Mk-model, potentially biasing the test in favour of the Mk-model. Furthermore, they did not consider whether the simulated data exhibited realistic levels of homoplasy, analysed unrealistically large simulated datasets, and evaluated only the relative performance of equal-weights parsimony when morphological data are now commonly analysed under implied-weights parsimony [6].

In an attempt to evaluate the relative performance of likelihood and parsimony methods for the phylogenetic analysis of discrete character morphological data, we simulated datasets of 100, 350 and 1000 discrete morphological characters using a modified HKY85 model, discriminating datasets that failed to meet expected levels of homoplasy. We evaluated the relative performance of equal-weights parsimony, implied-weights parsimony and model-based methods of phylogenetic analysis in terms of their ability to recover the tree used to simulate the data. We found that the Mk-model performs best in the analysis of all simulated datasets, largely because the Bayesian consensus trees are poorly resolved. Equal-weights parsimony exhibits lower levels of accuracy but this is combined with higher resolution. Implied-weights parsimony performed most poorly of all the methods considered.

## 2. Material and methods

To simulate binary morphological data, we used the HKY + *Γ*_continuous_ model to generate nucleotide data which we translated into purines (0) and pyrimidines (1)—R/Y coding. The recoded HKY-model possesses an uneven equilibrium distribution of state frequencies, resulting in structurally realistic morphological matrices while facilitating violation of assumptions of the Mk-model; thus, our data are not biased in favour of either method of phylogenetic inference. Initial tests were performed to determine values for the model parameters which produce binary data with empirically observed levels of homoplasy [7]. Following [5], data were simulated using the lissamphibian tree presented in [8], yielding datasets of 100, 350 and 1000 characters; most real morphological datasets contain in the order of 100 characters, but we included 350 and 1000 character matrices to investigate the effect of scaling and for ease of comparison to [5]. In total, 100 unique underlying substitution rates were drawn from a U(0.1,10) distribution, facilitating rates spanning two orders of magnitude. For each substitution rate, 10 unique matrices were produced, modelling among-character rate heterogeneity as gamma distributed uniquely within each matrix.

Matrices were analysed with the Mk + *Γ* model using default priors in MrBayes v. 3.2 [9], and both standard and implied-weights parsimony in TNT [10]. The Mk-model is more suitable for our simulated data than the Mkv-model as we did not strip invariant sites from the final matrices. Majority-rule consensus trees were produced for each method. For implied-weights parsimony, we used a range of *K*-values: 2, 3, 5, 10, 20 and 200. As the underlying substitution rate is varied, the per-matrix level of homoplasy may violate the empirically observed range; to produce the most empirically justified morphological matrices, we implemented an empirically derived minimum consistency index (CI) cut-off of 0.26 [7] for each simulated dataset and repeated analyses for these treated matrices (electronic supplementary material, figure S1). This cut-off reduced the size of the datasets to 128 (100 characters), 149 (350 characters) and 126 (1000 characters) matrices. In-depth description of the initial parameter value tests and further details of matrix generation are presented in the electronic supplementary material.

The accuracy of topologies estimated by the different reconstruction techniques was assessed using the Robinson–Foulds distance [11] from the generator tree. We also explored the relationship between resolution of output trees, measured by the number of nodes per tree.

## 3. Results

**Figure 1.**
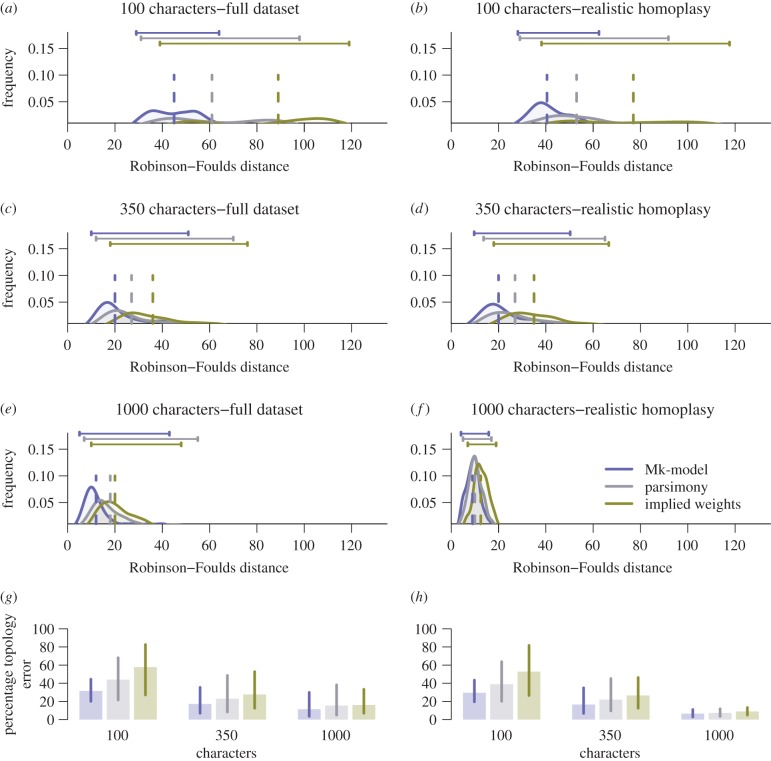
Mk tree reconstructions (blue) outperform equal-weights parsimony (grey) and implied-weights parsimony (green) for 100, 350 and 1000 characters (*a*,*c*,*e*,*g*), and these differences remain in the subset of the simulated data matrices that exhibit realistic levels of homoplasy (*b*,*d*,*f*,*h*). Bars above the plots mark the 95th percentile range for each method, and dashed vertical lines show the median values. Percentage topology error (*g*,*h*) is the Robinson–Foulds value of the reconstructed tree compared with the worst possible value, as shown in [5].

The Mk-model achieved the highest levels of accuracy across all datasets. Median Robinson–Foulds distances are lower for the Mk-model compared with both equal-weights and implied-weights parsimony (table 1 and figure 1), and for all approaches, accuracy of topology reconstruction increases with increasing dataset size. Furthermore, equal-weights parsimony out-performs implied-weights parsimony for all datasets and values of *K*, but this is less pronounced for the 1000 character dataset (table 1). For convenience, all further results for implied weights are for *K* = 2.

**Table 1.** The differences in median and the 95th percentile range of Robinson–Foulds values between the Mk and both parsimony models are greater in the full dataset compared with the realistic homoplasy subsets. mk, Bayesian Mk model; ew, equal-weights parsimony; iw, implied weights parsimony and its attendant *K* values.

	100 characters	100 characters CI	350 characters	350 characters CI	1000 characters	1000 characters CI
mk	45 (29–64)	40.5 (28.2–62.5)	20 (10–51)	19.5 (10.2–57.3)	19.5 (10.2–57.3)	11 (5–27.8)
ew	61 (31–98)	53 (29–91.8)	27 (12–70)	28 (12–74.8)	28 (12–74.8)	16 (6.2–43.7)
iw k2	89 (39–119)	77 (38.2–117.7)	36 (18–76)	36 (17.2–81.3)	36 (17.2–81.3)	19.5 (10–35.7)
iw k3	76 (38–112)	69 (36.4–108)	32 (16–69)	34 (15.2–70)	34 (15.2–70)	18 (9.2–35.7)
iw k5	68 (36–104)	61 (32.2–102)	30 (14–66)	31.5 (15.2–68)	31.5 (15.2–68)	18 (9–34)
iw k10	63 (34–100)	55.5 (32–98)	28 (13–68)	30 (15.2–69.7)	30 (15.2–69.7)	16 (8–34)
iw k20	64 (34–100)	53 (33–97.8)	28 (14–68)	30 (13.2–71.7)	30 (13.2–71.7)	17 (8–39.3)
iw k200	65 (34–100)	55 (32.2–97.7)	28 (14–72)	30.5 (15–76)	30.5 (15–76)	18 (8–44)

The same relative performance of the phylogenetic reconstruction methods is seen when considering only those datasets exhibiting realistic levels of homoplasy. The median Robinson–Foulds distance for the Mk-model is still lowest for each dataset, but the median and range of Robinson–Foulds distances for equal and implied-weights parsimony are closer to the distribution seen from the Mk-model (table 1 and figure 1). Additionally, for a given dataset, there is a similar Robinson–Foulds distance regardless of the reconstruction method employed (electronic supplementary material, figure S2). Unless otherwise stated, all subsequent results are from the subset of datasets exhibiting realistic levels of homoplasy.

**Figure 2.**
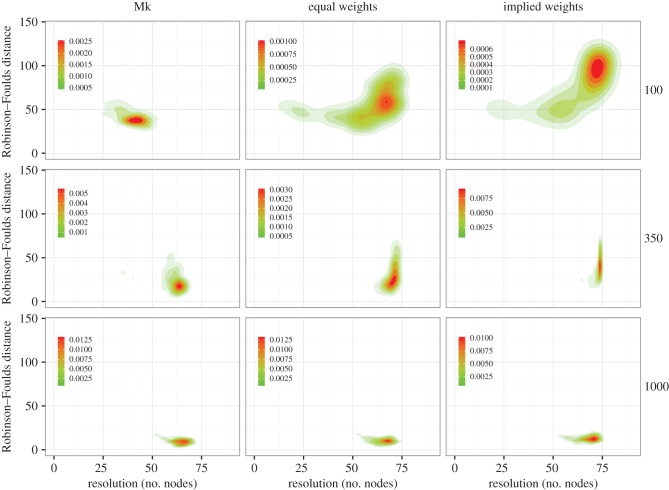
The Mk model exhibits higher accuracy with lower precision than parsimony methods; these results are less clear as more characters are added. Contour plots of Robinson–Foulds distances against the number of resolved nodes in each tree; the contours represent the density of the distribution of trees.

The higher accuracy (lower Robinson–Foulds values) of the Mk-model against other methods for 100 and 350 characters is due to trees being less resolved (figure 2). The density of Robinson–Foulds distance is lower for the Mk compared with equal weights, which itself is lower than implied weights, but both equal and implied weights achieve higher levels of precision (number of nodes reconstructed). These differences are negligible in the 1000 character datasets (figure 2).

There is a significant overlap in the set of nodes correctly recovered across methods, when mapped against the reference phylogeny (figure 2; electronic supplementary material, figure S3). In particular, for all methods there is a trend for nodes closer to the root to be more accurately estimated in small datasets, but this relationship decreases as the number of characters increases (electronic supplementary material, table S2 and figures S2, S4, S5). The percentage of times a node from the reference tree was accurately reconstructed showed a strong correlation for 100 and 350 characters, but decreases with 1000 characters (electronic supplementary material, table S2).

## 4. Discussion

Only minor differences are seen in the accuracy of phylogenetic topology reconstruction between the Bayesian implementation of the Mk-model and parsimony methods. Our findings both support and contradict elements of the results of Wright & Hillis [5] in that we can corroborate their observation, that the Mk-model outperforms equal-weights parsimony in accuracy, but the Mk-model achieves this at the expense of precision. Unexpectedly, implied-weights parsimony is less effective than either equal-weights parsimony or the Mk-model, in datasets with small numbers of characters. Implied-weights parsimony outperforms equal-weights parsimony only in the analyses of unrealistically large datasets. These results challenge the increasingly common view that implied-weighting better accommodates homoplasy than does equal-weights parsimony [6], and this result is true for a range of *K*-values (table 1).

In comparison with the other approaches, equal-weights parsimony analyses of the datasets exhibiting realistic levels of homoplasy and large number of characters yield a set of trees with a longer tailed distribution of Robinson–Foulds distances. In large part, this reflects estimation of a small quantity of trees markedly different from the generating tree (figure 1). Inaccuracy in topological estimation is more prevalent towards the tips in all analyses, with the inclusion of more characters reducing the intensity of this phenomenon. For this effect to be completely removed, it would require the analysis of well over 1000 empirically justifiable characters, a number that is rarely achieved for morphological datasets. The accuracy of node reconstruction is correlated significantly between all three techniques, demonstrating that most nodes in the tree that were difficult to resolve for one method were difficult to resolve for all. This phenomenon is observed across all character quantities and suggests a general difficulty in accurately estimating topology given the same data.

Our results can be interpreted to advocate use of the Mk-model over parsimony methods in the analysis of discrete morphological data. Parsimony methods produce precision without the accuracy achieved by the Mk-model and precision without accuracy is a poor basis for any science. We anticipate that the implementation of the Mk-model within a maximum-likelihood framework will exhibit levels of accuracy and precision more comparable to the parsimony methods, simply because it estimates a single, fully resolved topology. Integration over parameters while producing an acceptable level of accuracy is a quality of Bayesian inference, and our Mk-model results are probably dependent on a Bayesian implementation. While comparative phylogenetic methods often require fully resolved trees, these may be accommodated through analyses using the posterior sample of trees estimated using the Mk-model. Therefore, the prior requirement of a fully resolved tree need not necessarily lead to a preference for parsimony over the Mk-model.

In comparison to parsimony methods, the Mk-model has undergone little development since its conception [12,13], while attempts to improve the performance of parsimony methods, like implied-weights parsimony [3], have not led to increased accuracy (table 1). Thus, model-based phylogenetics can be expected to offer more opportunity for development, e.g. through relaxing the assumption of symmetrically distributed stationary distribution of character states [12,13] and improvement in the accuracy of phylogeny estimation from discrete character data. We suggest, however, that more focus should be invested in assessing whether the data are sufficiently informative to discriminate between competing phylogenetic hypotheses.

## 5. Conclusion

Phylogenies produced using likelihood models are more accurate than parsimony approaches, but have lower precision. Likelihood models offer greater scope for development in attempting to achieve greater accuracy but, in the interim, we suggest that phylogeneticists should consider the aims of their analyses when choosing the appropriate method.

## Supplementary Material

Electronic Supplementary Information

## Data accessibility

Data used in this manuscript are archived at http://dx.doi.org/10.5061/dryad.10qf3 and as the electronic supplementary material.
