# Supplementary material for: Bayesian methods outperform parsimony but at the expense of precision in the estimation of phylogeny from discrete morphological data
Source: Biol Lett. 2016 Apr;12(4):20160081. doi: 10.1098/rsbl.2016.0081 (PMC4881353; doi:10.1098/rsbl.2016.0081)
Supplement: Electronic Supplementary Information [file rsbl20160081supp1.docx]

**SUPPLEMENTARY INFORMATION**

**Supplementary Methods**

**Identifying substitution parameters that produce realistic levels of homoplasy**

To effectively determine the relative adequacy of the Mk-model and parsimony methodologies through simulated data it is important that the simulated matrices reflect the qualities of empirical matrices. To ensure our matrices contained data that resemble the empirical data for which these methods are usually employed we utilised homoplasy as a metric of empirical validity. Sanderson and Donoghue present a list of morphological matrices and their respective consistency index (CI) [1] . These matrices possess CIs that range from 0.26-1, providing a range within which our simulated matrices must fall to be considered empirically realistic. To identify optimal parameter values for the HKY+Γ_continuous_ model we simulated matrices whilst varying substitution model parameters and then retrieved CI scores for a number of unique replicates of each configuration of parameter values.

Nucleotide stationary frequency (π) was varied in increments of 0.05 such that all possible stationary frequencies between [A, G, C, T] = [0.45, 0.45, 0.05, 0.05] and [0.05, 0.05, 0.45, 0.45] were considered. When recoded as purine/pyrimidine this results in a vector of stationary frequencies ranging between [Pur, Pyr] = [0.9, 0.1] to [0.1, 0.9] with intermediate increments of 0.1 being considered.

We also considered 3 values for transition/transversion ratio (κ), 1, 3, and 5, which were combined with the varying values for π. Transversions were fixed to 1 and transitions varied such that they are expressed as a ratio against transversions, reducing the number of required parameters for simulation by one. The continuous gamma distribution under which between site heterogeneity was simulated was parameterised in such a way that the two standard parameters of the gamma distribution, shape and rate (α and β), were fixed to be equal. This further reduces the number of parameters required to vary for simulation by one and reflects the manner in which the composition of the gamma distribution is estimated in phylogenetic analyses. Setting α = β results in

$$E[X]=\frac{\alpha}{\beta}=1$$

For our simulated datasets this is helpful as it provides a sensible distribution of site-specific rates around the mean substitution rate, especially as site number increases where we expect the mean rate to be the underlying substitution rate. We explored the effect of varying α alongside π and κ on the resultant homoplasy in the character matrix through these initial simulations.

Under each combination of α, π, and κ, we simulated 1000 sites for 25 replicates and retrieved the CI scores for each replicate. We then evaluated the ability of each combination of parameter values to produce matrices with empirically observed levels of homoplasy by comparing the number of 25 replicates that possessed CIs that sat within the empirical range.

The number of replicates for each configuration of generating parameter values that possessed CIs within the empirically defined range are outlined in supplementary table S1.

Final parameter values used for the simulations based on the initial simulations were:

π = [0.15, 0.15, 0.35, 0.35] = [0.3, 0.7]

κ = 5

α = 5

All HKY+Γ simulations were built around the Phylosim package for R [2]. CIs were calculated using the phangorn package for R [3].

**References**

[1] Sanderson, M.J. & Donoghue, M.J. 1989 Patterns of variation in levels of homoplasy. *Evolution* **43**, 1781-1795.

[2] Sippos, B., Massingham, T., Jordan, G.E., Goldman, N. 2011 PhyloSim - Monte Carlo simulation of sequence evolution in the R statistical computing environment. BMC Bioinformatics 12:104

[3] Schliep K.P. 2011. phangorn: phylogenetic analysis in R. Bioinformatics, 27(4) 592-593

**Supplemetary Results**

|  | π=[0.1,0.9] | π=[0.2,0.8] | π=[0.3,0.7] | π=[0.4,0.6] | π=[0.5,0.5] | π=[0.6,0.4] | π=[0.7,0.3] | π=[0.8,0.2] | π=[0.9,0.1] |
| --- | --- | --- | --- | --- | --- | --- | --- | --- | --- |
| κ=1 α=0.5 | 0 | 0 | 0 | 0 | 0 | 0 | 0 | 0 | 0 |
| κ=1 α=2 | 2 | 0 | 0 | 0 | 0 | 0 | 0 | 0 | 1 |
| κ=1 α=5 | 2 | 0 | 0 | 0 | 0 | 0 | 0 | 0 | 1 |
| κ=3 α=0.5 | 25 | 0 | 0 | 0 | 0 | 0 | 0 | 0 | 25 |
| κ=3 α=2 | 25 | 23 | 0 | 0 | 0 | 0 | 0 | 24 | 25 |
| κ=3 α=5 | 25 | 25 | 4 | 0 | 0 | 0 | 2 | 25 | 25 |
| κ=5 α=0.5 | 25 | 23 | 0 | 0 | 0 | 0 | 0 | 22 | 25 |
| κ=5 α=2 | 25 | 25 | 25 | 25 | 20 | 25 | 25 | 25 | 25 |
| κ=5 α=5 | 25 | 25 | 25 | 25 | 25 | 25 | 25 | 25 | 25 |

**Supplementary Table S1:** The number of replicates, out of a possible 25, that possessed CI values within the empirically observed range.

| **Reconstruction method** | **Tree distance** | ***p value*** | **Nodes** | ***p value*** |
| --- | --- | --- | --- | --- |
| **Mk model (100 Characters)** | -0.329 | <0.001 | *-0.359* | <0.001 |
| **equal weights parsimony (100 Characters)** | -0.334 | <0.001 | *-0.368* | <0.001 |
| **implied weights (100 Characters)** | -0.4 | <0.001 | *-0.424* | <0.001 |
| **Mk model (350 Characters)** | -0.248 | 0.005 | *-0.28* | 0.002 |
| **equal weights parsimony (350 Characters)** | -0.274 | 0.002 | *-0.313* | <0.001 |
| **implied weights (350 Characters)** | *-0.327* | *<0.001* | *-0.357* | <0.001 |
| **Mk model (1000 Characters)** | -0.406 | <0.001 | *-0.477* | <0.001 |
| **equal weights parsimony (1000 Characters)** | -0.399 | <0.001 | *-0.467* | <0.001 |
| **implied weights (1000 Characters)** | *-0.183* | *0.043* | *-0.212* | 0.021 |

**Supplementary Table S2:** There is a tendency for nodes closer to the root to be more accurately estimated when measured using tree distance from the root or the number of nodes to the root. Correlation between percentage of the times nodes were accurately estimated and their distance from the root as measured by Kendall’s rank tau. Italic text indicates significant relationships.


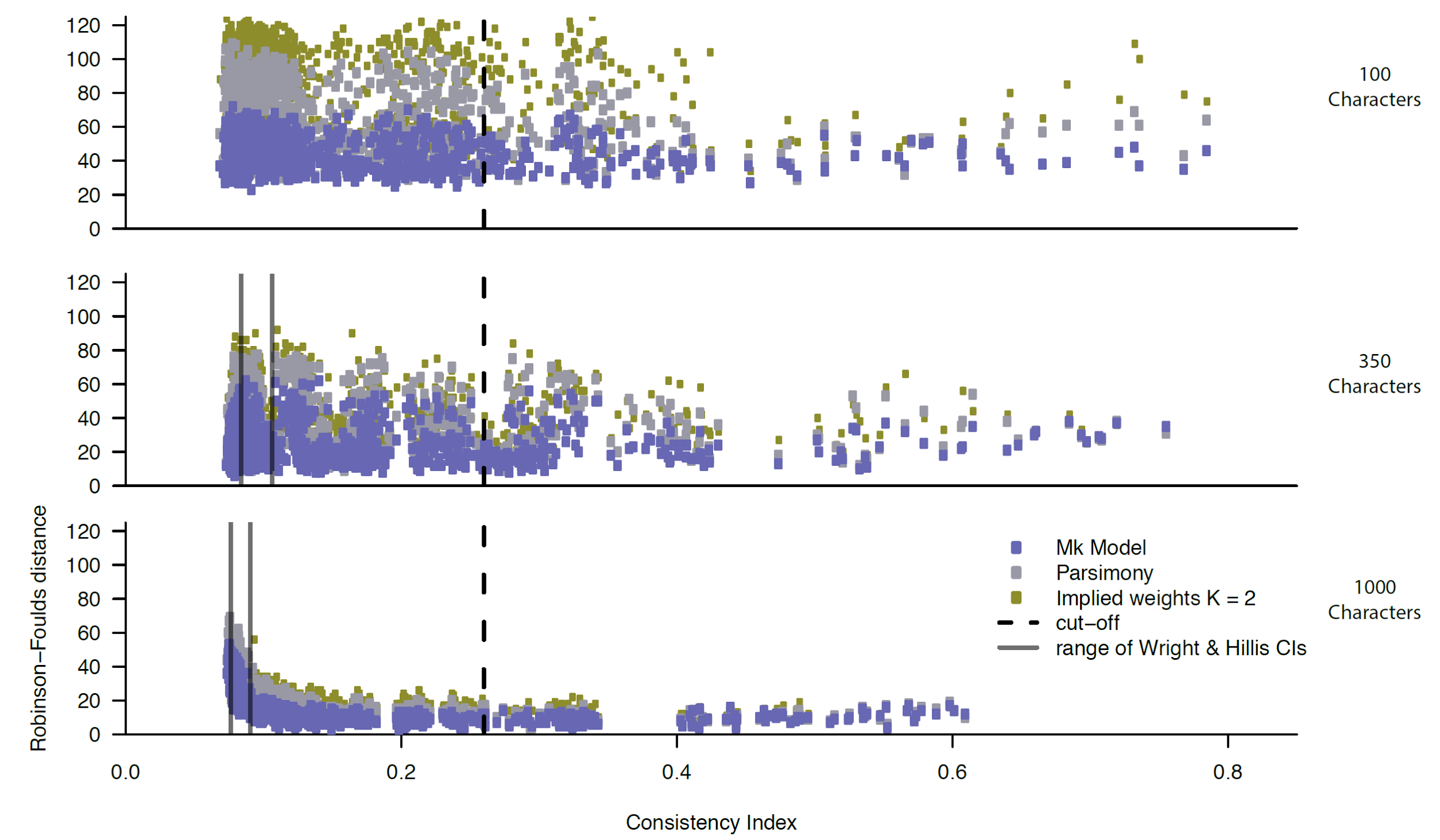


**Supplementary Figure S1:** Consistency Indices of the different datasets for the three phylogenetic reconstruction methods. The cut-off for the CI of 0.26 was selected and datasets below this figure were removed.


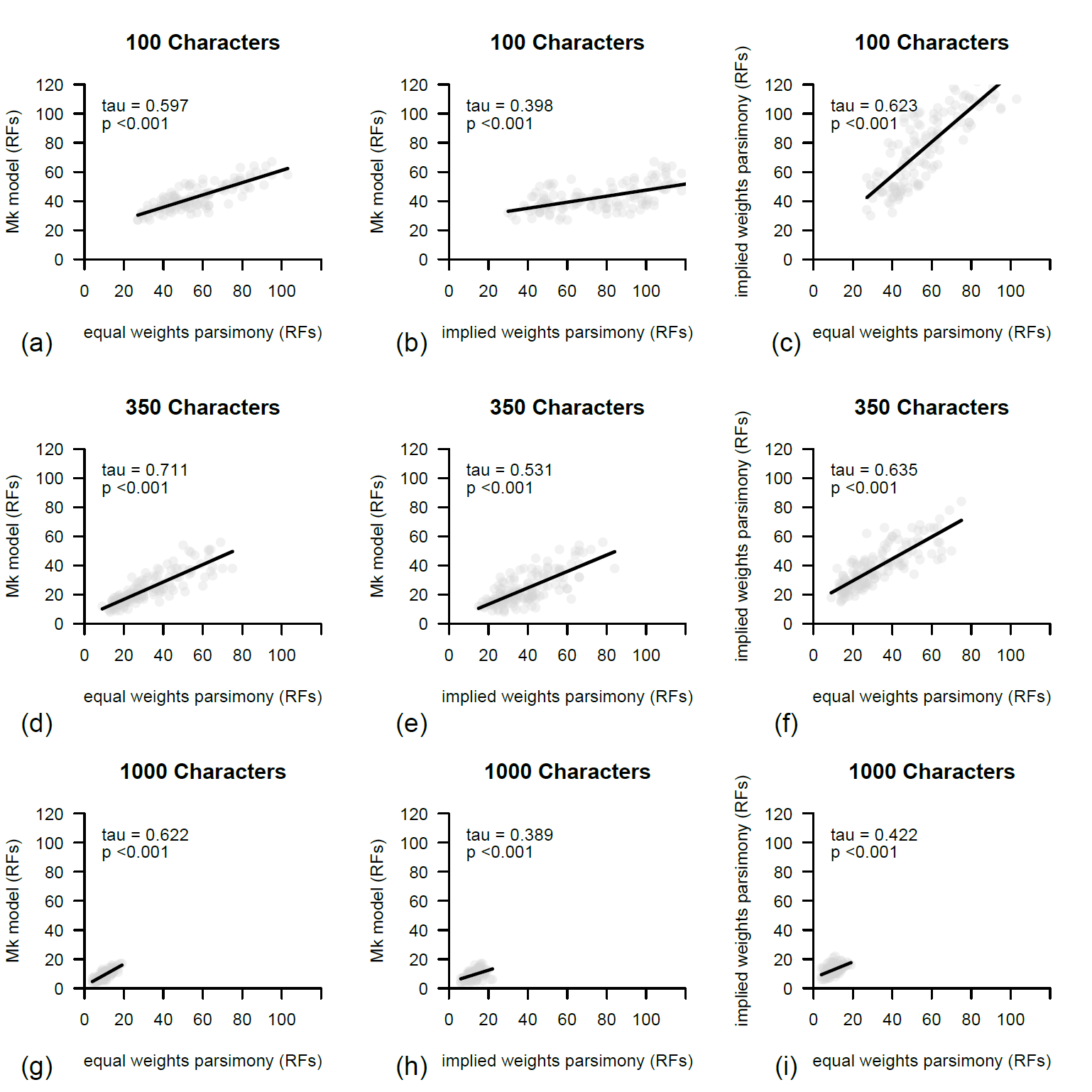


**Supplementary Figure S2.** For each individual dataset, there is a positive correlation between the Robinson-Foulds (RFs) accuracy for that dataset and the phylogenetic reconstruction method employed. This is show for equal weights parsimony and the Mk model (left) and implied weights and the Mk model (middle).


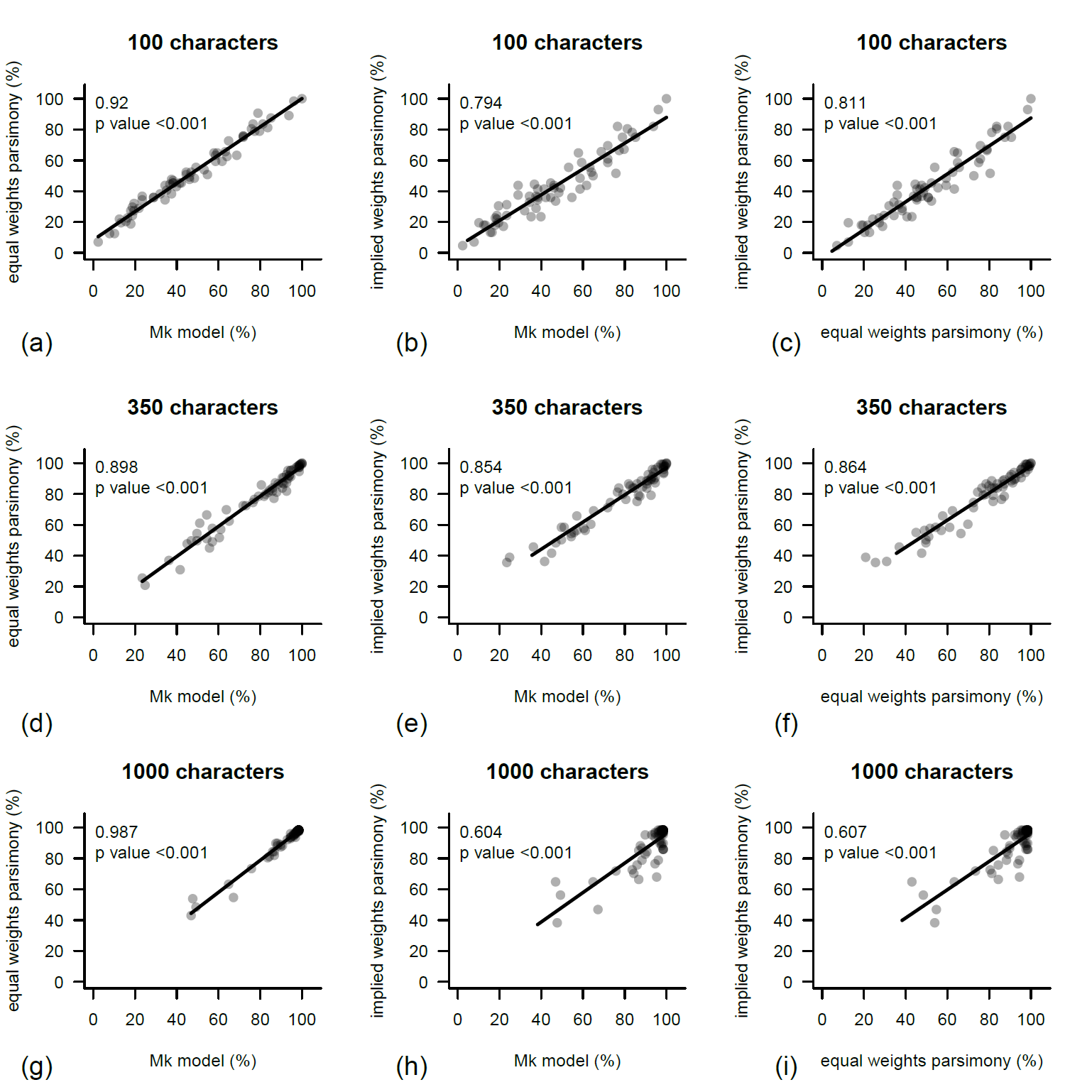


**Supplementary Figure S3:** The accuracy of node reconstructions is significantly correlated between the Mk with equal weights parsimony (left) and implied weights parsimony (left). Node accuracy is measure as the number of times a node from the simulation tree is accurately reconstructed in the 1000 reconstructed trees.


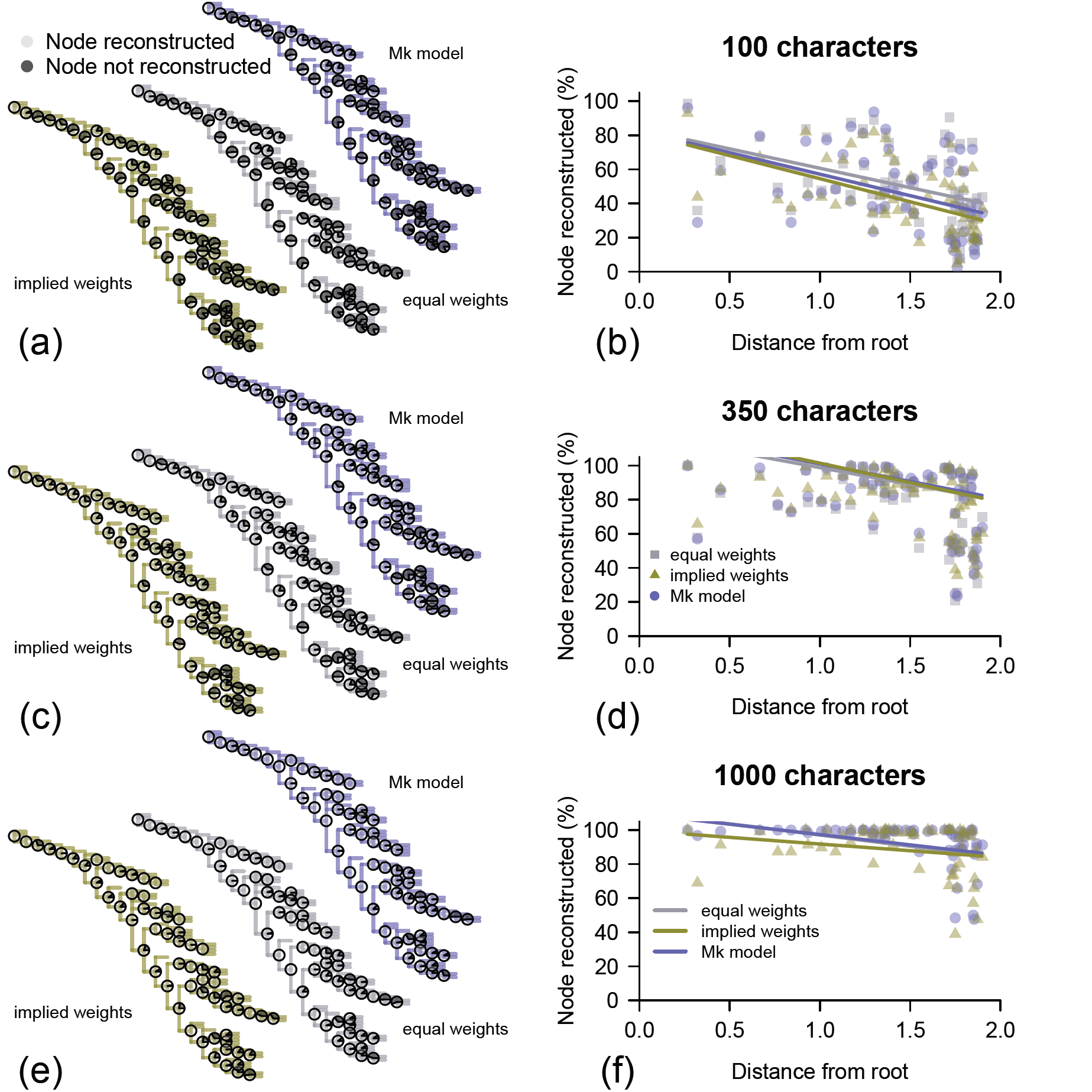


**Supplementary Figure S4:** The percentage of accurately reconstructed nodes (light grey) on the Mk trees, equal-weights, and implied-weights trees decreases with increasing distance from the node (a, b), but this relationship decreases for increasing dataset size (c, d, e, f).


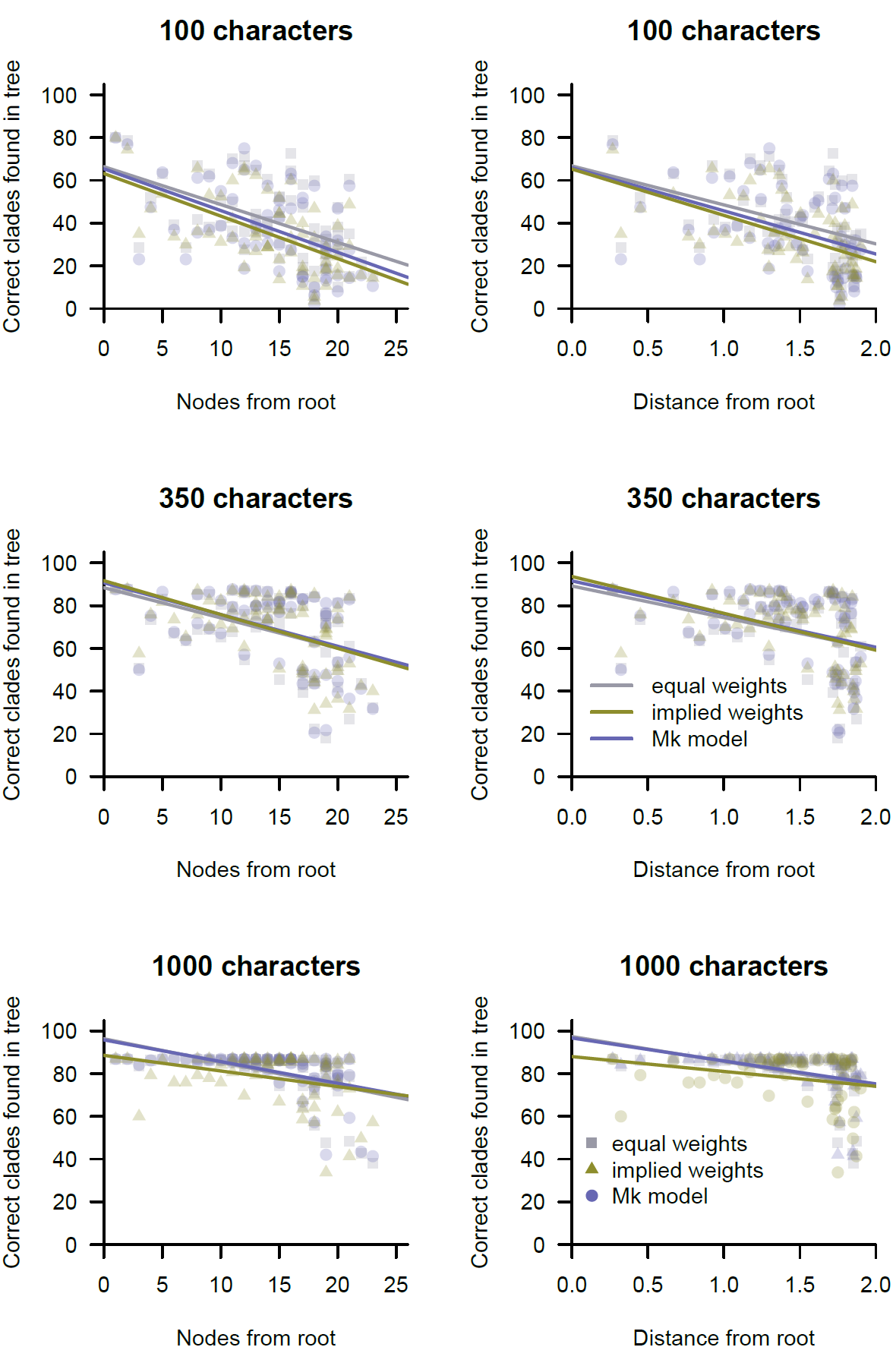
**Supplementary Figure S5:** There is a tendency for higher rates of error to be found further from the root for all trees when distance from the root is measured in the number of nodes (left) or branch lengths (right) for the Mk model (navy), parsimony (grey), and implied weights (green) reconstruction methods.
